# The effect of education participation on youth custody: Causal evidence from England

**DOI:** 10.23889/ijpds.v8i2.2216

**Published:** 2023-09-14

**Authors:** Sophie Dixon

**Affiliations:** 1 Instititute of Criminology, Univeristy of Cambridge, Cambridge, United Kingdom

## Objectives

The study aimed to evaluate the efficacy of victim age and co-offending status classification systems in distinguishing between diverse sexual offenders/offences. This can aid in enhancing comprehension of the correlates and aetiologies of these offences and inform the development of more effective criminal justice policy.

## Method

The current study uses Data First Ministry of Justice Crown Court Data from England and Wales between 2013 and 2019 to compare offender and offence characteristics between different typologies of sexual offending categorised using: (1) victim age (child sexual abuse v adult sexual abuse); (2) co-offending status (lone v duo v group); and (3) a combination of victim age and co-offending status (group child sexual abuse v group adult sexual abuse).

## Results

Significant differences between the typologies were found across a variety of variables for all three categorisation systems. These included the age, sex, ethnicity, and prosecution histories of offenders; age and ethnic composition of co-offending groups/duos; and the nature of the offences committed. While some findings support previous studies, some diverge from what has previously been reported in the literature. Furthermore, some of the findings provide information on areas which, until now, have received very little attention in the literature (i.e., comparisons of group perpetrated child sexual abuse and group perpetrated adult sexual abuse).

## Conclusion

This study provides support for the application of victim age and co-offending status classification systems to sexual offending as a means of disentangling heterogeneous groups of offenders/offences.

